# Horizontal Chromosome Transfer between Pathogenic and Non-pathogenic *Fusarium oxysporum* Strains Isolated from Cabbage

**DOI:** 10.1264/jsme2.ME25078

**Published:** 2026-04-25

**Authors:** Yu Ayukawa, Misaki Kaino, Takashi Yaeno

**Affiliations:** 1 Graduate School of Agriculture, Ehime University, 3–5–7 Tarumi, Matsuyama, Ehime, 790–8566, Japan; 2 Faculty of Agriculture, Ehime University, 3–5–7 Tarumi, Matsuyama, Ehime, 790–8566, Japan

**Keywords:** horizontal chromosome transfer, pathogenicity chromosomes, conidial anastomosis tube fusion

## Abstract

Horizontal chromosome transfer (HCT) has been demonstrated in *Fusarium oxysporum*. Several pathogenic *F. oxysporum* strains have been used as donors in HCT experiments, while the non-pathogenic strain Fo47 has mainly been employed as a recipient. It currently remains unknown whether other non-pathogenic *F. oxysporum* strains are recipients of mobile chromosomes. In the present study, we investigated whether the non-pathogenic strain 08C-3B, obtained from cabbage, acquired the mobile chromosomes of *F. oxysporum* f. sp. *conglutinans* strain Cong:1-1, which infects cabbage. We detected HCT between Cong:1-1 and 08C-3B in a conidial anastomosis tube (CAT) fusion-inductive medium, yielding HCT progeny strains that carried scaffolds (SCs) 8 and 9 of Cong:1-1. These progeny strains exhibited reduced colony growth on potato dextrose agar plates and produced no symptoms on cabbage. These results suggest that SC8 and/or SC9 hinder vegetative growth, but do not confer virulence to 08C-3B. We then conducted HCT experiments to assess whether the HCT progeny strain transfers the acquired chromosomes to other strains. However, no progeny strains were obtained, suggesting that 08C-3B does not function as a donor for mobile chromosomes.

The soil-borne pathogenic fungus *Fusarium oxysporum* infects over 100 plant species. Due to the limited host range of each pathogenic strain, these strains may be classified into more than 100 formae speciales (ff. spp.) based on their host plants and symptoms. The pathogenicity chromosomes associated with host specificity have been identified in some ff. spp. of *F. oxysporum* (Ma *et al.*, 2010; van Dam *et al.*, 2017; Li *et al.*, 2020; Ayukawa *et al.*, 2021). The tomato-infection pathogen *F. oxysporum* f. sp. *lycopersici* (Fol) strain 4287 has a single pathogenicity chromosome enriched in effector-encoding genes, such as secreted in xylem (*SIX*) genes (Ma *et al.*, 2010). Tomatoes infected with Fol4287 mutant strains lacking this chromosome do not develop symptoms (Vlaardingerbroek *et al.*, 2016b). Similarly, cucurbit-infecting *F. oxysporum* f. sp. *radicis-cucumerinum* strain 016 possesses a pathogenicity chromosome that confers virulence in cucumber, melon, and watermelon (van‍ ‍Dam *et al.*, 2017). However, the crucifer-infecting *F. oxysporum* f. sp. *conglutinans* strain Cong:1-1 has multiple pathogenicity chromosomes (Ayukawa *et al.*, 2021). Its genome consists of 22 scaffolds (SCs). A comparative genomic anal­ysis revealed that the SC10/SC20 or SC16/SC18 set constituted individual chromosomes, designated as chr^SC10/SC20^ and chr^SC16/SC18^, respectively. HS5, a mutant strain of Cong:1-1 lacking SC9 and chr^SC10/SC20^, exhibited reduced virulence toward *Arabidopsis thaliana.* The gene *SIX4* on SC9 is associated with virulence in *A. thaliana* and cabbage (Thatcher *et al.*, 2012; Kashiwa *et al.*, 2013). The effectors, *SIX8* and *PSE1*, found on chr^SC10/SC20^, are involved in virulence toward *A. thaliana* (Ayukawa *et al.*, 2021). Another mutant HS6 lacking SC5, SC8, SC9, and chr^SC10/SC20^ exhibited reduced virulence toward *A. thaliana* and cabbage. *SIX1* on SC8 was shown to be a full virulence factor in cabbage (Li *et al.*, 2016). Therefore, SC8, SC9, and chr^SC10/SC20^ are regarded as pathogenicity chromosomes. The presence of multiple pathogenic *F. oxysporum* strains may be attributed to the diversification of pathogenicity chromosomes carrying effector-encoding genes.

Non-pathogenic strains may become pathogenic due to the horizontal transfer of pathogenicity chromosomes (Ma *et al.*, 2010; van Dam *et al.*, 2017; Li *et al.*, 2020). These chromosomes are transferable between *F. oxysporum* strains. The non-pathogenic *F. oxysporum* isolate, Fo47, has been used in horizontal chromosome transfer (HCT) experiments as a recipient. Although Fo47 protects some plants from pathogens (Veloso and Díaz, 2012), its progeny strains carrying pathogenicity chromosomes exhibit virulence in tomato or cucurbits (Ma *et al.*, 2010; Vlaardingerbroek *et al.*, 2016a; van Dam *et al.*, 2017; Li *et al.*, 2020). Heterokaryon formation and nuclear fusion have been shown to occur between donor and recipient strains via conidial anastomosis tube (CAT) fusion (Shahi *et al.*, 2016). CAT fusion suppresses vegetative incompatible reactions, resulting in heterokaryon formation (Ishikawa *et al.*, 2012). CAT fusion is not observed in potato dextrose broth, but occurs under carbon-starved and nitrogen-limiting conditions (Shahi *et al.*, 2016). Despite its inhibitory effects, potato dextrose agar (PDA) is mainly used to co-cultivate donor and recipient strains (van der Does and Rep, 2012). Other non-pathogenic *F. oxysporum* strains that acquire pathogenicity chromosomes under these conditions, similar to Fo47, have yet to be identified.

The non-pathogenic *F. oxysporum* strain 08C-3B was first isolated from cabbage (Kashiwa *et al.*, 2013). A phylogenetic anal­ysis using ribosomal DNA intergenic spacer sequences showed that 08C-3B did not belong to the *F. oxysporum* f. sp. *conglutinans* clade (Kashiwa *et al.*, 2013). HCT from *F. oxysporum* strains has yet to be reported in 08C-3B. We herein investigated whether 08C-3B acquired mobile chromosomes from Cong:1-1. We found that (1) 08C-3B obtained SC8 and SC9 from Cong:1-1 on CAT-inducing medium, but not on PDA medium, and (2) the transferred chromosomes inhibited vegetative growth, but did not confer virulence to 08C-3B. We also exami­ned whether the mobile chromosomes received by 08C-3B were transferred to other strains. No HCT progeny strains were obtained, suggesting that 08C-3B was not a suitable donor for transferring the chromosomes received.

## Materials and Methods

### Fungal strains

Cong:1-1 wild-type, the Cong:1-1-derived mutant Δ*SIX4* (Kashiwa *et al.*, 2013), the Δ*SIX4*-derived mutant HS6 lacking SC5, SC8, SC9, and chr^SC10/SC20^ (Ayukawa *et al.*, 2021), and the non-pathogenic strain 08C-3B (Kashiwa *et al.*, 2013) were used. These strains were precultured on PDA plates (AS ONE) at room temperature. To harvest microconidia, agar pieces containing mycelia were incubated in NO_3_ medium (0.17% [w/v] yeast nitrogen base [YNB] without amino acids, 3% [w/v] sucrose, and 100 mM KNO_3_) at 120 strokes min^–1^ (spm) and 28°C for 3 d. Regarding single-spore isolation, 10 μL of autoclaved water was added to the surface of a fungal colony grown on a PDA plate supplemented with hygromycin and G418, and the resulting conidia-containing suspension was collected. The suspension was then spread on a PDA plate and incubated at 28°C. After 3–5 d of incubation, a single colony derived from an individual spore was isolated.

### Growth assay

Fungal strains were precultured on PDA at room temperature for 3 d. Agar plugs containing mycelia were immersed in NO_3_ and incubated at 120 spm for 3 d. After harvesting microconidia, 10 μL of the microconidial suspension (1.0×10^6^ conidia mL^–1^) was inoculated on a PDA plate and incubated at 28°C in the dark. Each experiment included five plates. Colony diameters were measured 5 d after the incubation.

Cellophane penetration assays were conducted based on a previous study (Palos-Fernández *et al.*, 2022) with slight modifications. Briefly, an autoclaved cellophane sheet (TOYO) was placed on a PDA plate supplemented with 0.1 M Tris-HCl (pH 8.0). The sheet was inoculated with 10 μL of the microconidial suspension (2.5×10^6^ conidia mL^–1^) and incubated at 28°C in the dark for 3 d. The sheet was then removed and the plate was incubated at 28°C in the dark for 1 d.

### Infection assay

A cabbage cultivar, Tokinashi (Atariya Noen), was grown in Supermix A (Sakata Seed) under 12/12 h light/dark conditions at 28°C. Four-day-old plants were irrigated with 1 mL of the conidial suspension (1.0×10^6^ conidia mL^–1^). Each pot contained four plants. Before the inoculation, the roots of the plants were injured with a peg. The shoot weight of the cabbage plant was recorded at 14 d post-inoculation. To observe root colonization by *F. oxysporum*, cabbage roots were collected at 10 d post-inoculation and then exami­ned using the fluorescence microscope, BZ-X810 (Keyence).

### Plasmid construction

To construct pGWB1-GG, *nptII* and *EGFP* expression cassettes were amplified from pMD-GEN (Saito *et al.*, 2021) using the KOD One PCR Master Mix (TOYOBO) with the primers P_11 and P_12. To construct pGWB1-RZ containing the *ble* and *DsRED2* expression cassettes, the *ble* cassette was amplified from the genomic DNA of HS6-ble (Ayukawa *et al.*, 2021) with the P_122 and P_123 primers. The *DsRED2* expression cassette was amplified from pAK2-HYG (Kato *et al.*, 2012) with the P_12 and P_136 primers. The vector backbone for both plasmids was amplified from pGWB1 (Nakagawa *et al.*, 2007) using the P_13 and P_14 primers. The resulting fragments were ligated using the In-Fusion HD Cloning Kit (TaKaRa Bio) or NEBuilder HiFi DNA Assembly Master Mix (New England Biolabs).

### Agrobacterium tumefaciens-mediated transformation (ATMT)

Cong:1-1 wild-type, Δ*SIX4*, HS6, and 08C-3B were transformed via ATMT as previously described (Takken *et al.*, 2004; Dong and Wang, 2022) with some modifications. *A. tumefaciens* EHA105 carrying pGWB1-GG or pGWB1-RZ was incubated in 2YT medium supplemented with 25 μg mL^–1^ kanamycin and 25 μg mL^–1^ rifampicin at 120 spm and 28°C for 24 h. Five milliliters of the suspension was diluted to an OD_600_ of 0.45 with the induction medium (IM) and centrifuged at 1,800×*g* for 5 min. After discarding the supernatant, the pellet was resuspended in 5 mL of IM supplemented with 400 μg mL^–1^ acetosyringone (AS) and incubated at 28°C for 6 h. Three milliliters of the microconidial suspension (2.0×10^5^ conidia mL^–1^) was mixed with 3 mL of the bacterial cell suspension. One milliliter of this mixture was then spread on a filter paper placed on the co-cultivation medium supplemented with 400 μg mL^–1^ AS and incubated at 28°C. After 2 d of co-incubation, the filter papers were transferred onto PDA plates supplemented with 285 μg mL^–1^ cefotaxime sodium salt and 300 μg mL^–1^ G418 or 100 μg mL^–1^ zeocin and incubated at 28°C for 2 d. After removing the filter paper, the fungal colonies on PDA plates were isolated as transformants.

### HCT experiment

HCT experiments were performed as previously described (van der Does and Rep, 2012). Briefly, the microconidial suspensions (10^6^ conidia mL^–1^) of the donor and recipient strains were mixed at a 1:1 (v/v) ratio, and 800 μL of this mixture was then spread on four plates containing PDA or CAT-inducing medium (CAT medium) (0.17% [w/v] YNB without amino acids, 25 mM KNO_3_, and 1.5% [w/v] agar). The plates were incubated at 28°C for 7 d. Mycelia and conidia were harvested by adding 5 mL of sterile water to the plates and disrupting the colonies with a cell spreader. The mycelial suspension containing conidia was spread on PDA supplemented with 100 μg mL^–1^ hygromycin and 300 μg mL^–1^ G418 or PDA containing 0.1 M Tris-HCl (pH 8.0), and 100‍ ‍μg‍ ‍mL^–1^ hygromycin and 100 μg mL^–1^ zeocin. A single hyphal tip of each double drug-resistant colony was transferred to a PDA plate supplemented with the antibiotics. Mycelia or DNA were used as templates for PCR to verify HCT.

### PCR

PCR was performed using a 10-μL reaction mix containing 1× KOD One PCR Master Mix (TOYOBO), 0.3 μM of each primer, and 50 ng of fungal genomic DNA with the PCR Thermal Cycler Dice (TaKaRa Bio). The primers used in this study are listed in Table S1. PCR conditions were as follows: 30 cycles of denaturation at 98°C for 10 s, annealing at 60°C for 5 s, and extension at 68°C for 10 or 15 s. PCR products were separated on a 1% (w/v) agarose gel containing ethidium bromide by electrophoresis and visualized under UV light.

### Reverse transcription (RT)-PCR

To isolate RNA, mycelia were harvested from 3-d-old fungal strains grown in NO_3_ medium by filtering with Kimwipe (Nippon Paper Crecia). Trapped mycelia were dried with a paper towel and placed in a screw tube containing zirconia beads. Mycelia were then frozen with liquid nitrogen and crushed using the Shake Master NEO (Biomedical Science). Total RNA was extracted from disrupted mycelia using the RNeasy Plant Mini Kit for RNA Extraction (QIAGEN). cDNA was synthesized from 1 μg of total RNA using the PrimeScript RT reagent Kit with gDNA Eraser (TaKaRa Bio), and 10 ng cDNA was used for PCR as described above.

### CAT fusion assay

To observe CAT fusion, microconidia of the donor and recipient strains were suspended in CAT liquid medium, and 1 mL of each‍ ‍suspension (7.5×10^5^ conidia mL^–1^) was mixed. This mixture was incubated in a Petri dish with a special polymer bottom (Biomedical Science) at 28°C for 14–118 h in the dark. CAT fusion‍ ‍was observed under the fluorescent microscope, BZ-X810 (Keyence).

### Statistical anal­ysis

Statistical anal­yses were conducted using the R software package. The Kruskal-Wallis test and Dunn’s test with the Bonferroni correction were performed using the rcompanion (version 2.4.21) and FSA packages (version 0.10.0).

## Results

### HCT between Cong:1-1 and 08C-3B

**Fig. 1.**
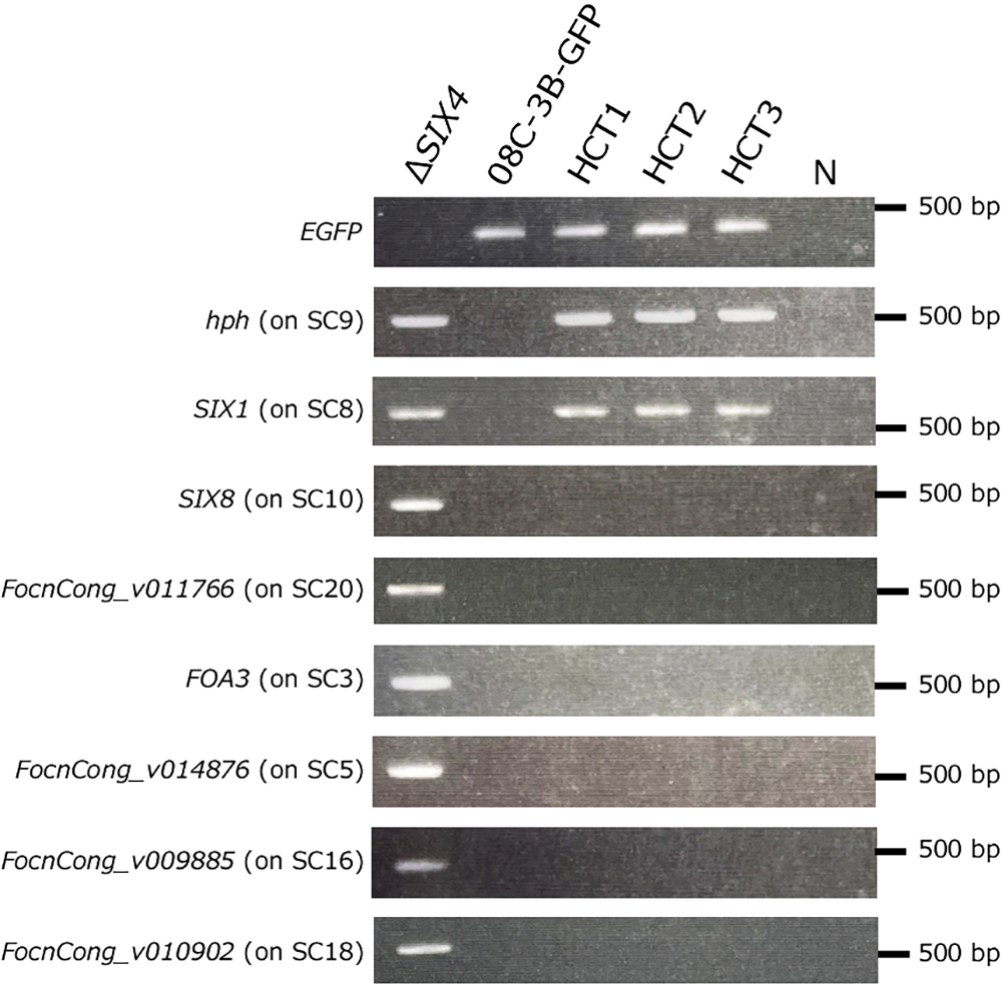
PCR-based detection of transferred chromosomes in horizontal chromosome transfer progeny strains derived from 08C-3B. The marker genes *EGFP*, *hph* (located on SC9), *SIX1* (on SC8), *SIX8* (on SC10), *FocnCong_v011766* (on SC20), *FOA3* (on SC3), *FocnCong_v014876* (on SC5), *FocnCong_v009885* (on SC16), and *FocnCong_v010902* (on SC18) were amplified from the genomic DNA of Δ*SIX4*, 08C-3B-GFP, and HCT progeny strains (HCT1, HCT2, and HCT3). Lane N indicates a water control.

PDA is commonly used to co-cultivate donor and recipient *F. oxysporum* strains in HCT experiments (Ma *et al.*, 2010; Vlaardingerbroek *et al.*, 2016a; van Dam *et al.*, 2017; Li *et al.*, 2020). To investigate whether 08C-3B acquired the mobile chromosomes from *F. oxysporum* f. sp. *conglutinans*, we co-cultivated G418-resistant 08C-3B-GFP with hygromycin-resistant Δ*SIX4* of Cong:1-1, which contains the hygromycin-resistance gene *hph* on SC9, as previously described (van der Does and Rep, 2012). The conidia and mycelia were harvested from the co-cultivation plates and plated on selective media containing hygromycin and G418. We performed HCT experiments six times and obtained more than 20 double drug-resistant colonies. However, PCR assays showed that *hph* was not detected in any colonies (data not shown), suggesting that these colonies were false positives. Since CAT fusion is known to mediate HCT (Shahi *et al.*, 2016) and is induced under nutrient-poor conditions, we co-cultivated Δ*SIX4* with 08C-3B-GFP on the CAT-inducing medium developed by Shahi *et al.* (2016). In one out of three independent experiments, 24 double drug-resistant colonies were obtained on selective plates. The PCR anal­ysis confirmed the presence of the *EGFP*-encoding gene and *hph* in the genomic DNA of three colonies (Fig. 1). To verify HCT, molecular marker genes specific to the dispensable chromosomes of Cong:1-1 were amplified, which were detected in the donor strain, but not in the original recipient (Fig. 1). In the double drug-resistant strains, the molecular marker genes for SC9 and SC8 were detected, whereas those for SC3, SC5, chr^SC10/SC20^, and chr^SC16/SC18^ were not (Fig. 1). These results confirmed that the double drug-resistant colonies were HCT progeny strains that acquired SC8 and SC9. To further investigate the presence of additional scaffolds in the remaining 21 colonies, colony PCR assays were performed with primer sets targeting marker genes for dispensable chromosomes. Although the marker genes for SC8 and SC9 were detected, those for SC3, SC5, chr^SC10/SC20^, and chr^SC16/SC18^ were not (data not shown). EGFP fluorescence was observed in all 21 colonies under the fluorescence microscope. To examine the stability of SC8 and SC9 in HCT1–3, a single spore was isolated from each HCT progeny and incubated on a PDA plate. The PCR anal­ysis detected *SIX1* on SC8 and *hph* on SC9 in the genomic DNA (Fig. S1), indicating that SC8 and SC9 were stably maintained in HCT1–3.

### Effects of mobile chromosomes on 08C-3B

**Fig. 2.**
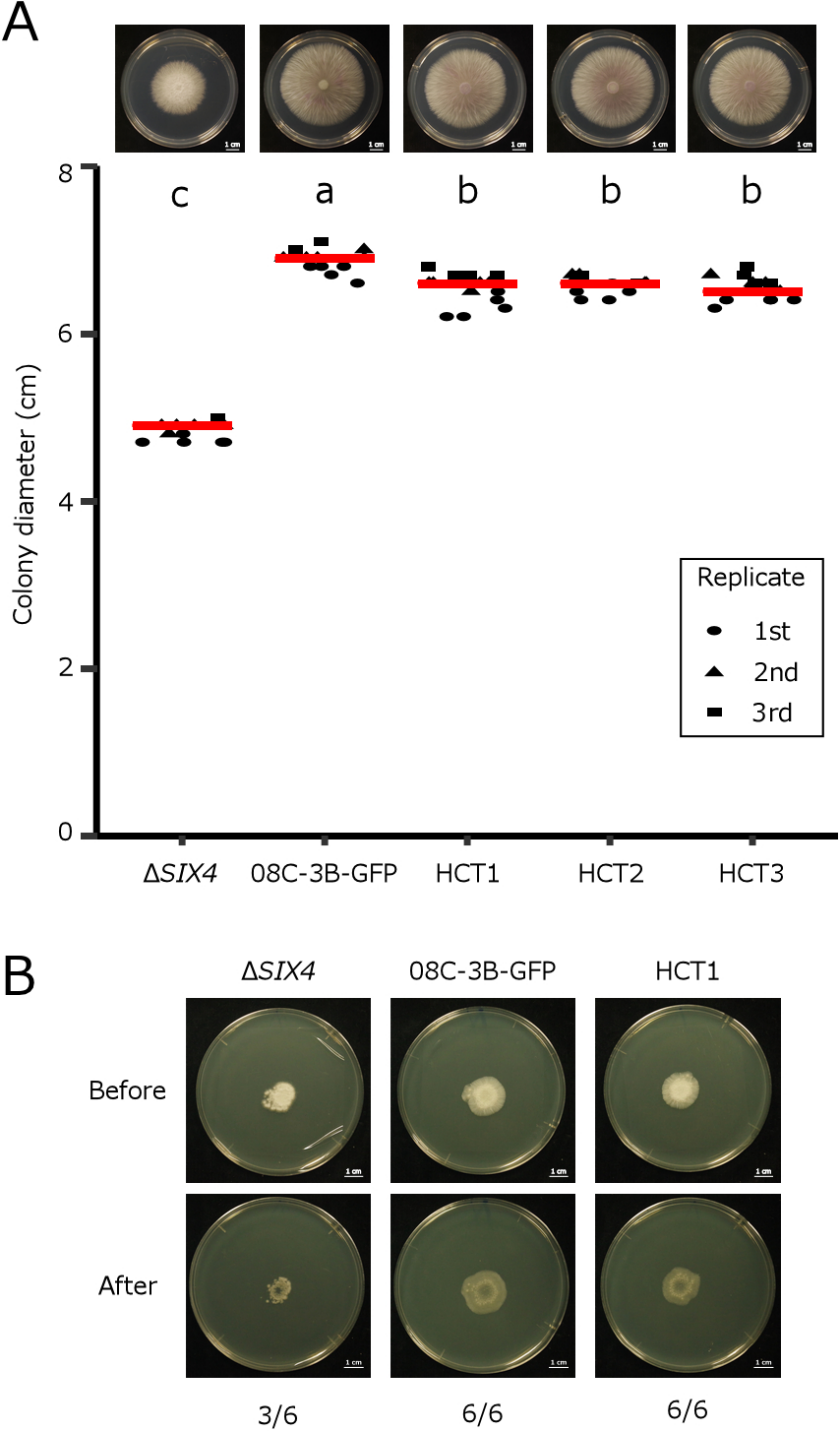
Transferred chromosomes affect the colony growth of 08C-3B, but not its invasive growth. (A) Colony morphology and diameter of Δ*SIX4*, 08C-3B-GFP, and HCT progeny strains grown on potato dextrose agar (PDA). Five-day-old colonies were photographed (top panels). The dot plot shows the colony diameters measured in three independent experiments (bottom). Each experiment included five plates, and the symbols represent individual colonies from each experiment (1st: circle, 2nd: triangle, 3rd: square; *n*=15). Red bars indicate the average diameters. Different letters above the dotplots indicate significant differences in the Kruskal-Wallis test followed by Dunn’s test (*P*<0.05). (B) The invasive growth of Δ*SIX4*, 08C-3B-GFP, and HCT1. Colonies were grown on or under cellophane sheets placed on PDA supplemented with 100 mM Tris-HCl (pH 8.0) and photographed before and after removing the sheets, respectively. The number of plates showing cellophane-penetrating colonies out of six plates is indicated below each image.

To examine the effects of the transferred chromosomes on the vegetative growth of 08C-3B, we compared the colony morphologies of the HCT progeny strains grown on PDA plates with those of the parental strains. The donor strain Δ*SIX4* developed a small whitish colony, while the recipient 08C-3B colonies exhibited a purplish color around the middle (Fig. 2A). Although the colony morphology of the HCT progeny strains closely resembled that of 08C-3B, the colony diameter of these progeny strains was significantly smaller than that of the recipient strain (Fig. 2A).

We next investigated whether the transferred chromosomes affected the invasive growth of 08C-3B. Previous studies demonstrated that pathogenic *F. oxysporum* strains penetrated cellophane sheets (Prados Rosales and Di Pietro, 2008; Liu *et al.*, 2019). To test this, we incubated Δ*SIX4*, 08C-3B-GFP, and HCT1 on cellophane sheets placed on PDA for 3 d. After removing the cellophane sheets, Δ*SIX4* colonies were observed on three out of six plates (Fig. 2B). 08C-3B and HCT1 colonies penetrated the sheets on all plates, indicating that SC8 and SC9 did not affect the invasive growth of 08C-3B (Fig. 2B). These results suggest that the transferred chromosomes negatively regulated radial growth, but did not affect invasive growth in 08C-3B.

### Effects of SC8 and SC9 on the virulence of 08C-3B

**Fig. 3.**
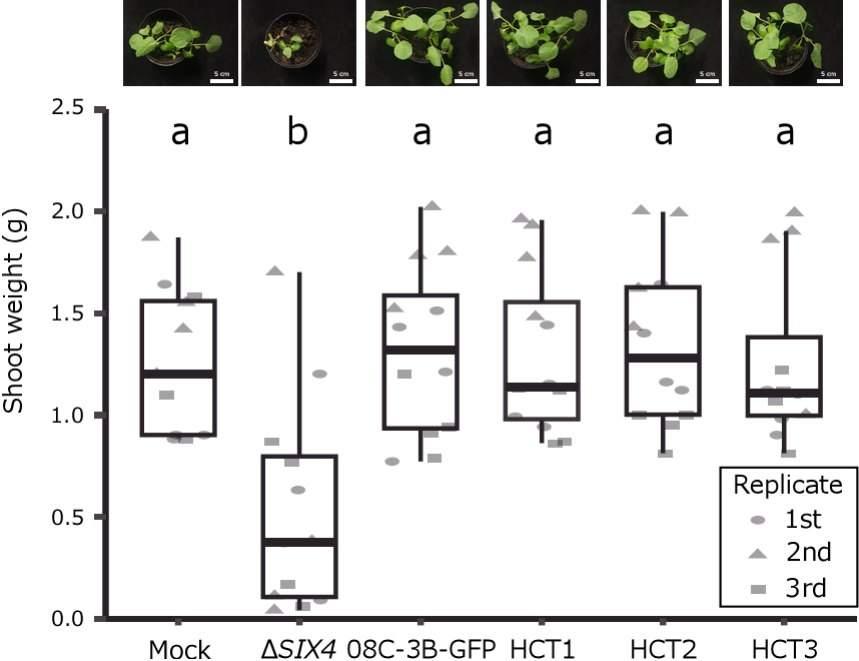
SC8 and SC9 do not confer virulence on cabbage to 08C-3B. Four-day-old cabbage plants were inoculated with water, Δ*SIX4*, 08C-3B-GFP, or HCT progeny strains. The plants were photographed at 14 d post-inoculation (top panels). Boxplots show shoot weights measured in three independent experiments (bottom), indicating the median (thick line), 25th and 75th percentiles (box hinges), and 1.5× the interquartile range (whiskers). The symbols represent individual plants from each experiment (1st: circle, 2nd: triangle, 3rd: square, *n*=12). Different letters above the plots indicate significant differences in the Kruskal-Wallis test followed by Dunn’s test (*P*<0.05).

**Fig. 4.**
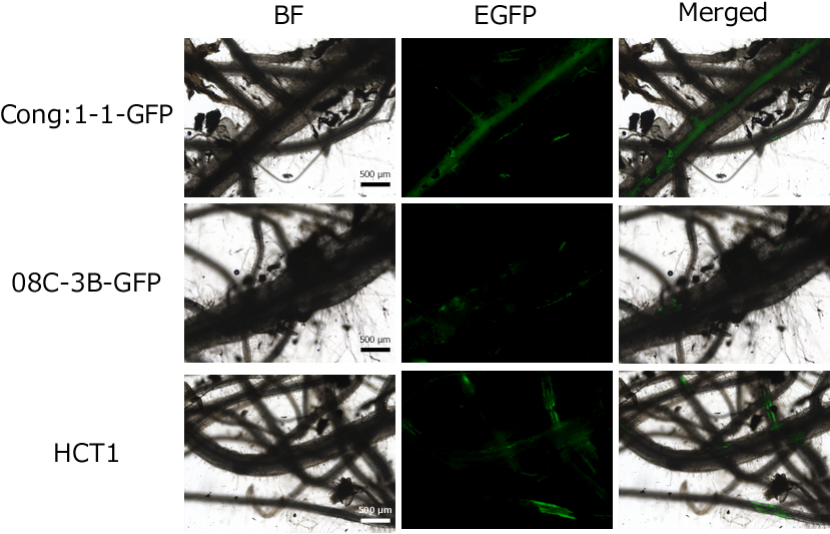
SC8 and SC9 do not affect root colonization by 08C-3B. Four-day-old cabbage plants were inoculated with Cong:1-1-GFP, 08C-3B-GFP, or HCT1. Cabbage roots were exami­ned under a fluorescent microscope at 10 d post-inoculation. Representative images are shown in brightfield (left), fluorescence (middle), and merged channels (right).

To investigate the virulence of the HCT progeny strains, we inoculated cabbage with the Δ*SIX4*, 08C-3B-GFP, and HCT progeny strains. While Δ*SIX4* markedly reduced the shoot weight of cabbage, the 08C-3B-GFP and HCT progeny strains did not exhibit any virulence or impact on the growth of cabbage (Fig. 3). To assess whether SC8 and SC9 affected root colonization by 08C-3B, we observed cabbage roots inoculated with EGFP-labeled strains under the fluorescent microscope. *EGFP*-expressing Cong:1-1 colonized and proliferated within the stele of the main root, whereas 08C-3B-GFP and HCT1 were restricted to the root surface and did not invade the stele (Fig. 4).

**Fig. 5.**
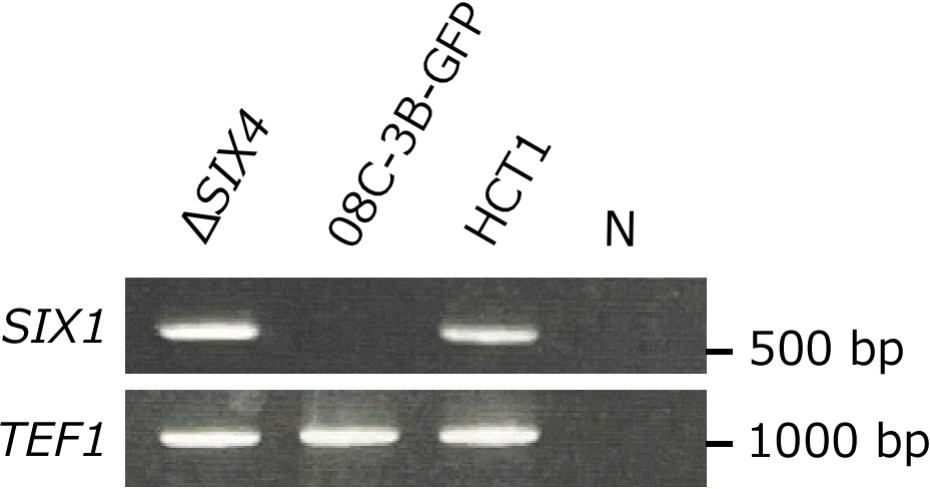
*SIX1* on a transferred chromosome in HCT1 is transcribed. *SIX1* transcription was analyzed by RT-PCR. PCR was performed using 10 ng of cDNAs obtained from total RNA extracted from the mycelia of Δ*SIX4*, 08C-3B-GFP, and HCT1 grown in NO_3_ medium. This experiment was repeated twice with similar results. Translation elongation factor 1α (*TEF1*) was amplified as an internal control.

The effector gene *SIX1*, located on SC8, is a full virulence factor for cabbage, which is highly expressed *in vitro* and *in planta* (Li *et al.*, 2016). To confirm *SIX1* expression in HCT1, we conducted RT-PCR using cDNA synthesized from RNA extracted from hyphae developed in NO_3_, which induces effector gene expression. *SIX1* was transcribed in HCT1, similar to that in Δ*SIX4* (Fig. 5).

### SC9 is not transferred from HCT1 to other strains

Previous studies (Ma *et al.*, 2010; van Dam *et al.*, 2017; Li *et al.*, 2020) demonstrated that pathogenic *F. oxysporum* strains transfer mobile chromosomes to the non-pathogenic strain. However, it remains unknown whether HCT progeny strains transfer the acquired chromosome to other strains. To assess the HCT capability of the progeny strain HCT1, we generated a zeocin-resistant HS6-RED, a Cong:1-1-derived mutant lacking pathogenicity chromosomes, using *A. tumefaciens* EHA105 carrying pGWB1-RZ, and conducted HCT experiments using HCT1 and HS6-RED as the donor and recipient strains, respectively. The co-cultivation of HCT1 with HS6-RED resulted in the formation of abundant aerial hyphae on the selective plates. To isolate the double drug-resistant strains, conidia were harvested and spread on a fresh selective plate. Five colonies that formed on the plate were subjected to colony PCR to detect the presence of drug-resistance genes. However, all colonies carried either *hph* or the zeocin-resistant gene *ble* (Fig. S2A). Although we repeated the experiment six times, we were unable to confirm HCT between HCT1 and HS6-RED. To investigate whether HCT1 introduced SC9 into 08C-3B, we generated zeocin-resistant 08C-3B (08C-3B-RED) and used it as the recipient strain. Similar to HS6, abundant aerial hyphae were generated on the selective plates after spreading the mycelia and conidia harvested from the co-cultivation plates. To confirm HCT, we performed PCR to detect *hph* and *ble* from 10 colonies isolated from the selective plates. *ble* was detected in all colonies, whereas *hph* was not (Fig. S2B), suggesting that all colonies were false positives. Although we conducted the experiments six times, we were unable to obtain HCT progeny.

**Fig. 6.**
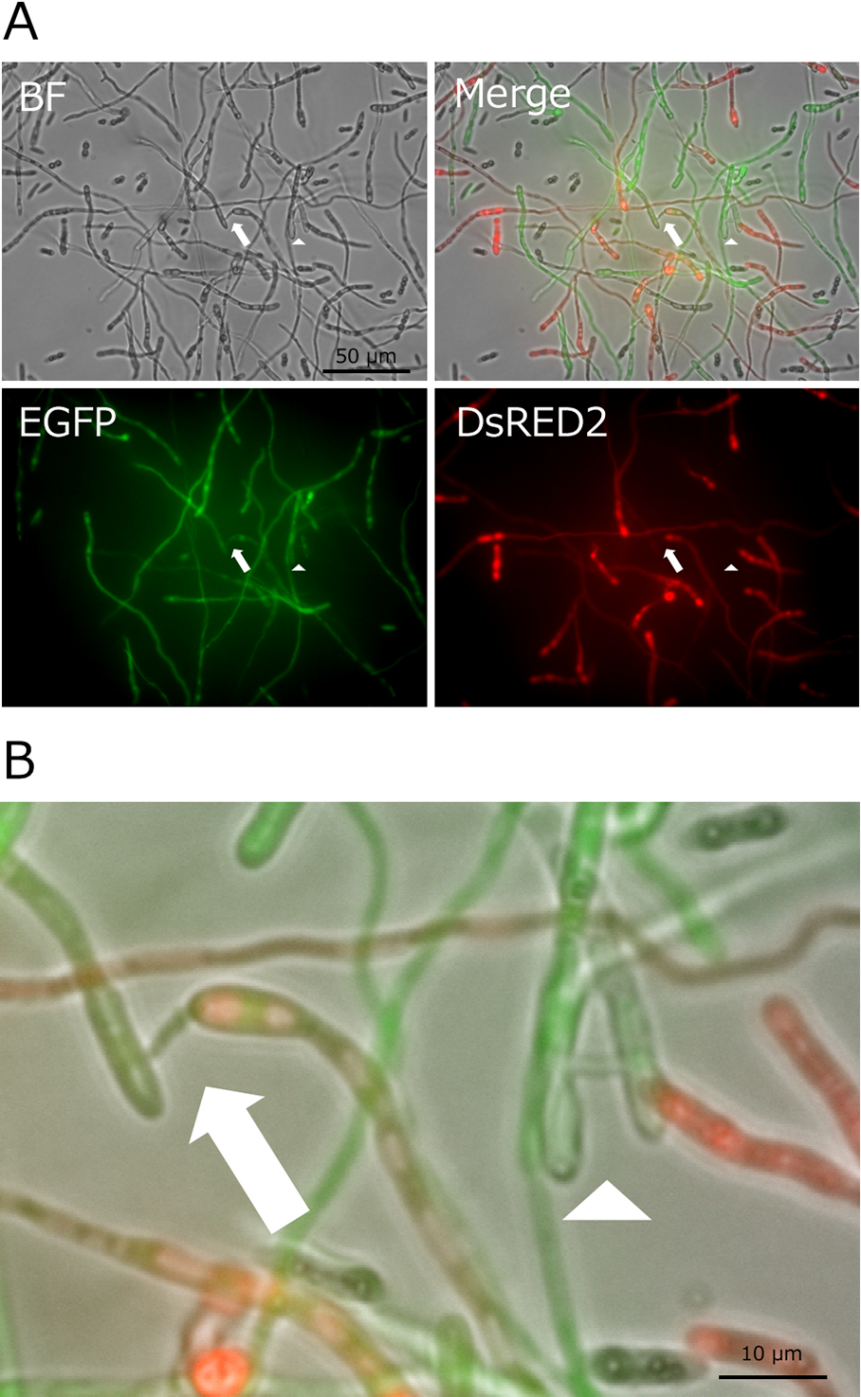
Conidial anastomosis tube (CAT) fusion induces cytoplasmic mixing between HCT1 and 08C-3B-RED. (A) HCT1 (green) and 08C-3B-RED (red) were co-incubated in CAT-inducing liquid medium (CAT liquid medium). CAT fusion was exami­ned under a fluorescence microscope at 14–16 h post-incubation. Arrows indicate CAT fusion between HCT1 and 08C-3B-RED, and arrowheads indicate CAT fusion between HCT1 hyphae. Representative images are shown in brightfield (BF), EGFP, DsRED2, and merged channels. A magnified image of CAT fusion is shown in (B).

To investigate whether CAT fusion occurred between HCT1 and other strains, HCT1 was co-incubated with HS6-RED or 08C-3B-RED in CAT liquid medium for 14–19 h. CAT structures were observed between HCT1 and 08C-3B-RED. Furthermore, EGFP and DsRED2 signals were detected in hyphae, indicating successful cytoplasmic fusion (Fig. 6). Although CAT structures were observed between HCT1 and HS6-RED at 118 h post-incubation, cytoplasmic fusion was not detected (Fig. S3). As a control, we co-incubated 08-3B-RED with Δ*SIX4* expressing *EGFP* in the CAT liquid medium. However, we did not observe cytoplasmic fusion through CAT structures (Fig. S4). These results suggest that SC9 is not transferable in 08C-3B even if cytoplasmic fusion occurs.

## Discussion

We herein demonstrated that the non-pathogenic *F. oxysporum* strain 08C-3B isolated from cabbage acquired SC8 and SC9 from Cong:1-1 on CAT medium. However, HCT progeny strains from 08C-3B did not form on PDA plates. We previously showed that SC8, SC9, and chr.^SC10/SC20^ of Cong:1-1 may be complemented in HS6 by the co-cultivation of HS6 with Δ*SIX4* on PDA (Ayukawa *et al.*, 2021). Although PDA is generally used for chromosome transfer or complementation experiments, CAT fusion was not induced in potato dextrose broth (Shahi *et al.*, 2016; Kurian *et al.*, 2018), indicating that PDA is unsuitable for HCT experiments. In *Verticillium dahliae*, CAT fusion and heterokaryosis between strains of different vegetative compatibility groups or species were induced under starvation conditions (Vangalis *et al.*, 2021). Therefore, nutrient-poor conditions may induce CAT fusion and HCT in specific *F. oxysporum* isolates, such as 08C-3B, allowing HCT progeny strains to be generated from various non-pathogenic *F. oxysporum* isolates. However, we cannot exclude the possibility of HCT between Cong:1-1 and 08C-3B on PDA because the number of experimental trials is still limited.

To the best of our knowledge, this is the first study to report the effects of HCT on the vegetative growth of recipient *F. oxysporum* strains. We found that SC8 and/or SC9 of Cong:1-1 inhibited the radial growth of 08C-3B on PDA (Fig. 2A). Furthermore, the vegetative growth of HS1, an SC9-deficient mutant of Cong:1-1, was similar to that of wild-type Cong:1-1 (Ayukawa *et al.*, 2021), suggesting that SC8 carried the genes that negatively regulate vegetative growth. However, the transferred chromosomes did not affect the invasive growth of 08C-3B (Fig. 2B). Although Δ*SIX4* colonies occasionally failed to penetrate cellophane sheets, the 08C-3B and HCT1 colonies consistently penetrated the sheets. The penetration of *F. oxysporum* colonies into cellophane sheets has been linked to virulence (Prados Rosales and Di Pietro, 2008). However, cabbage inoculated with 08C-3B did not exhibit any growth inhibition. In the case of Cong:1-1 and 08C-3B, this capability may not always elicit virulence in cabbage. Fluorescent microscopy revealed that 08C-3B colonized the epidermis of cabbage roots (Fig. 4). Since this strain was isolated from cabbage (Kashiwa *et al.*, 2013), 08C-3B may be an endophytic isolate that exhibits invasive growth to colonize the root epidermis. While Cong:1-1 invaded xylem tissues and propagated therein, 08C-3B failed to penetrate xylem vessels (Fig. 4). It may be possible to confirm whether a strain is a cabbage pathogen or endophyte by its ability to penetrate and colonize vascular tissues. *SIX1*, an established virulence factor on cabbage, is highly expressed *in vitro* and *in planta* (Li *et al.*, 2016). We demonstrated that *SIX1* on SC8 was expressed in the hyphae of HCT1 (Fig. 5). Since HCT1 colonized the root surface, but not the xylem, *SIX1* may not be associated with xylem invasion. The Six1 protein has been detected in the xylem sap of infected cabbage (Pu *et al.*, 2016); therefore, this gene may be involved in xylem colonization. Furthermore, SC3 has been associated with virulence in cabbage (Ayukawa *et al.*, 2021). Since SC3 is not transferred to 08C-3B, HCT progeny strains lack the virulence genes that cause disease in cabbage. SC3 retains two putative effector-encoding genes, *SIX9* and *FOA3* (Ayukawa *et al.*, 2021). Foa3 has been shown to suppress the chitin-induced production of reactive oxygen species, whereas Six9 did not (Tintor *et al.*, 2020). Therefore, Foa3 potentially suppresses pattern-triggered immunity responses in cabbage roots, which may be required for xylem penetration.

Although the CAT medium allowed 08C-3B to receive the mobile chromosomes, the HCT progeny strain was unsuccessful in introducing SC9 into HS6 or 08C-3B in the medium (Fig. S2). Therefore, 08C-3B may not be able to transfer mobile chromosomes. Given the low HCT efficiency in 08C-3B, the capacity to receive mobile chromosomes may be associated with the ability to transfer the chromosomes to others. HCT has been linked with CAT fusion, heterokaryosis, nuclear fusion, and the loss of donor core chromosomes (Shahi *et al.*, 2016). Since CAT fusion between HCT1 and 08C-3B was observed (Fig. 6), it is possible that heterokaryosis or nuclear fusion did not occur. In contrast, CAT fusion was not detected between HCT1 and HS6 (Fig. S3). CAT fusion was also not found between Δ*SIX4* with 08-3B in our assays (Fig. S4); therefore, it remains unclear whether CAT fusion occurs between HCT1 and HS6. The efficiency of CAT fusion may depend on the fungal strain pairs, even if CAT structures are formed. To the best of our knowledge, evidence for HCT progeny strains providing transferred chromosomes to other strains is limited. Further studies are needed to confirm whether non-pathogenic *F. oxysporum* strains transfer mobile chromosomes.

## Citation

Ayukawa, Y., Kaino, M., and Yaeno, T. (2026) Horizontal Chromosome Transfer between Pathogenic and Non-pathogenic *Fusarium oxysporum* Strains Isolated from Cabbage. *Microbes Environ ***41**: ME25078.

https://doi.org/10.1264/jsme2.ME25078

## Supplementary Material

Supplementary Material
