# Supplementary material for: Horizontal Chromosome Transfer between Pathogenic and Non-pathogenic Fusarium oxysporum Strains Isolated from Cabbage
Source: Microbes Environ. 2026 Apr 25;41(2):ME25078. doi: 10.1264/jsme2.ME25078 (PMC13293700; doi:10.1264/jsme2.ME25078)
Supplement: Supplementary file 1 — Supplementary Material [file 41_25078_s1.pdf]

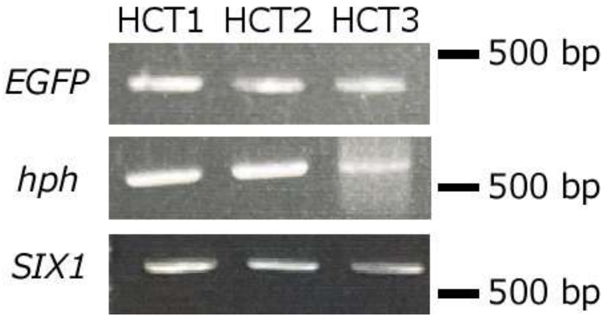

Fig. S1 SC8 and SC9 are stably maintained in HCT progeny after a single-spore isolation.

Marker genes, *EGFP*, *hph* (located on SC9), and *SIX1* (on SC8) were amplified from the genomic DNA of single-spored HCT progeny strains (HCT1, HCT2, and HCT3).

Yu Ayukawa: E-mail: ayukawa.yu.di@ehime-u.ac.jp;

TEL: +81-89-946-9810.

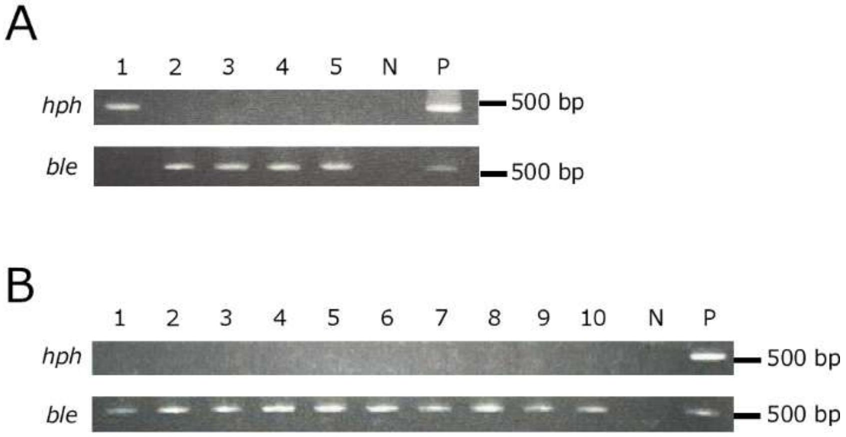

Fig. S2 HCT1 does not transfer SC9 to HS6-RED and 08C-3B-RED.

Colony PCR-based detection of *hph* and *ble* genes in double drug-resistant colonies generated by coincubation of HCT1 with HS6-RED (A) or 08C-3B-RED (B). *hph* and *ble* were amplified from the mycelia of double drug-resistant colonies generated from HCT1 co-cultivated with HS6-RED (lanes 1–5 in A) or 08C-3B-RED (lanes 1–10 in B). P indicates pMK412 containing *hph* or genomic DNA of HS6-ble carrying *ble*. N indicates water.

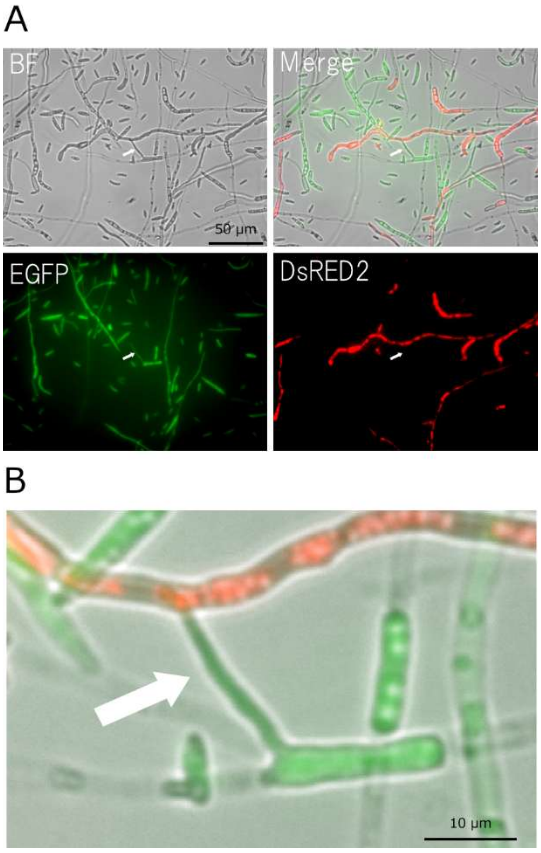

Fig. S3 Co-cultivation of HCT1 with HS6-RED in CAT liquid medium.

(A) HCT1 (green) and HS6-RED (red) were coincubated in CAT liquid medium. A CAT structure was observed at 118 h post-incubation. Arrows indicate a CAT structure formed between HCT1 and HS6-RED. Representative images are shown in brightfield (BF), EGFP, DsRED2, and merged channels. A magnified image of the CAT fusion is shown in (B).

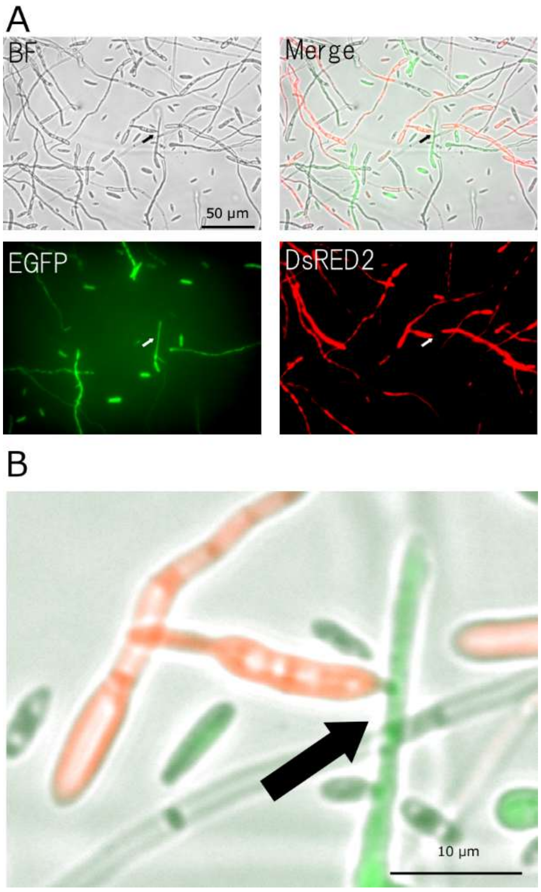

Fig. S4 Co-cultivation of 08C-3B-RED with Δ*SIX4* expressing *EGFP* in CAT liquid medium.

(A) Δ*SIX4* expressing *EGFP* (green) and 08C-3B-RED (red) were coincubated in CAT liquid medium. A CAT structure was observed at 118 h post-incubation. Arrows indicate a CAT structure formed between 08C-3B-RED and Δ*SIX4* expressing *EGFP*. Representative images are shown in brightfield (BF), EGFP, DsRED2, and merged channels. A magnified image of the CAT fusion is shown in (B).

Table S1. Primers used in this study

Primer name	Purpose	Sequence 5'-3'	Reference
P_11	Construction of pGWB1-GG (amplification of *nptII* and *EGFP* cassettes)	GGGAAACGACAATCTCTAGAAAGAAGGATTACCTC	This study
P_12	Construction of pGWB1-GG and pGWB1-RZ (amplification of *nptII* and *EGFP* cassettes or *DsRED2* cassette)	GCTTCTAGGACGCGTCTGTCTGGTCTTCTACACG	This study
P_136	Construction of pGWB1-RZ (amplification of *DsRED2* cassette)	AACTGCTAGCCTTAGGGATCCGGGTAGC	This study
P_122	Construction of pGWB1-RZ (amplification of *ble* cassette)	AAGGCGGGAAACGACAATCTTCTAGAAAGAAGGATTACCTCTAAAC	This study
P_123	Construction of pGWB1-RZ (amplification of *ble* cassette)	TTGCTACCCGGATCCCTAAGGCTAGCAGTTCCAGGTGG	This study
P_13	Construction of pGWB1-RZ (amplification of a backbone vector)	ACGCGTCCTAGAAGCTAATTCACT	This study
P_14	Construction of pGWB1-RZ (amplification of a backbone vector)	AGATTGTCGTTTCCCGCCTT	This study
GFP_F	Detection of *EGFP*	CACGACTTCTTCAAGTCCGC	This study
GFP_R	Detection of *EGFP*	TGTTGTAGTTGTACTCCAGCTT	This study
A5'	Detection of *hph*	TCTCGATGAGCTGATGCTTTGG	Zhang et al., 2000
B3'	Detection of *hph*	AGTACTTCTACACAGCCATCGG	Zhang et al., 2000
3655	Detection of *ble*	ATGACCGAGATCGGCGAGCA	Ayukawa et al., 2021
3656	Detection of *ble*	ACATGCAATTATCTTTGCGAACCC	Ayukawa et al., 2021
SIX8F	Detection of *SIX8*	GTGGCTGCTACGCATATTGA	Ayukawa et al., 2021
SIX8R	Detection of *SIX8*	TCGTGTACCGCTTGTGAGAG	Ayukawa et al., 2021
FOA3_F	Detection of *FOA3*	AGTCTGATGTCCCTCCTCCT	This study
FOA3_R	Detection of *FOA3*	CTGCTGGGCACTGGTTATGT	This study
SC16_F	Detection of *FocnCong_v009885*	GTCCTTGCTACCACCACCTT	This study
SC16_R	Detection of *FocnCong_v009885*	TCAACGACCAGAACTCAAGGT	This study
SIX1F	Detection of *SIX1*	CTCTCAATCCTTGGGTTTGG	Ayukawa et al., 2021
SIX1R	Detection of *SIX1*	TCCCCTCTCCACTCGAATAA	Ayukawa et al., 2021
Fc_v014876_fw	Detection of *FocnCong_v014876*	AGGTTGCTAAAGGTCGAGGC	This study
Fc_v014876_rv	Detection of *FocnCong_v014876*	TTAGTCTCGATGCTCACGGC	This study
18	Detection of *FocnCong_v011766*	ATGGTACAGGGCCAATG	Ayukawa et al., 2021

19	Detection of *FocnCong_v011766*	TCAAGACTTATTACGCTTCTCTTG	Ayukawa et al., 2021
SC18_F	Detection of *FocnCong_v010902*	ATGATATCGACAGGCTCCATA	This study
SC18_R	Detection of *FocnCong_v010902*	TATTGTCGAGAAGTTCCTGAC	This study
EF_forward2	Detection of *TEF1*	GCTACTATGTCACCGTCATTGACGCTCC	This study
EF_reverse3	Detection of *TEF1*	CGAAACGACCCAGAGGAGGGTAGTCAG	This study
